# Missing knowledge of gendered power relations among non-governmental organisations doing right to health work: a case study from South Africa

**DOI:** 10.1186/s12914-018-0172-4

**Published:** 2018-08-30

**Authors:** Mayara Fontes Marx, Leslie London, Alex Müller

**Affiliations:** 1 Icahn School of Medicine at Mount Sinai, New York, USA; 2 School of Public Health and Family Medicine, Faculty of Health Sciences, University of Cape Town, Cape Town, South Africa; 3 Gender Health and Justice Research Unit, University of Cape Town, Cape Town, South Africa

**Keywords:** Civil society organisations, Gender invisibility, Gender inequality, Health and human rights, Gender, women’s health

## Abstract

**Background:**

Despite 20 years of democracy, South Africa still suffers from profound health inequalities. Gender roles and norms are associated with individuals’ vulnerability that lead to ill-health. For instance, gender inequality influences women’s access to health care and women’s agency to make health-related decisions. This paper explores gender-awareness and inclusivity in organisations that advocate for the right to health in South Africa, and analyses how this knowledge impacts their work?

**Methods:**

In total, 10 in-depth interviews were conducted with members of The Learning Network for Health and Human Rights (LN), a network of universities and Civil Society Organisations (CSOs) which is explicitly committed to advancing the right to health, but not explicitly gendered in its orientation.

**Results:**

The results show that there is a discrepancy in knowledge around gender and gendered power relations between LN members. This discrepancy in understanding gendered power relations suggests that gender is ‘rendered invisible’ within the LN, which impacts the way the LN advocates for the right to health.

**Conclusions:**

Even organizations that work on health rights of women might be unaware of the possibility of gender invisibility within their organisational structures.

**Electronic supplementary material:**

The online version of this article (10.1186/s12914-018-0172-4) contains supplementary material, which is available to authorized users.

## Background

Despite 20 years of formal democracy, South Africa remains a country with profound inequalities in health status [1, 2] and in the distribution of resources needed for health along racial and gender lines [3, 4]. Ataguba and colleagues [5] confirmed a disproportionate burden from major categories of ill-health and disability amongst South Africans of low socio-economic status who also experience difficulties in access to primary care and hospital services [6].

However, South Africa is also a country with a rich tradition of civil society advocacy for human rights [7]. Strong civil society pressure was part of the political transition to democracy, including helping to shape the new South African constitution [3]. Subsequently, concerted civil society advocacy in health succeeded in turning around state AIDS denialism under president Thabo Mbeki, largely through an active citizenry using human rights as an educational, legal and mobilising tool to secure access to treatment for people living with HIV in South Africa [7–10]. Human rights non-governmental organisations (NGOs) thus were able to translate the constitutional promise of equality and dignity into real gains for ordinary people in relation to access to healthcare in South Africa, using human rights as a tool for citizens’ advocacy and activism [11].

Underlying health inequalities are varying degrees of powerlessness that render communities and individuals vulnerable to factors that lead to ill-health [12]. One of these social determinants of health is gender [13], and the resulting gendered power relations in patriarchal societies. According to the World Health Organization [14], *gender* refers to ‘the socially constructed roles, behaviours and activities and attributes that a given society considers appropriate for men and women’; while *sex* refers to biological differences between person classified as female or male. To improve health outcomes of individuals, it is important to understand the differences between the two concepts of ‘sex’ and ‘gender’. Biological differences attributed to sex generate specific health needs based on these biological differences (for example, the need for cervical cancer prevention for people with cervixes). However, there should also be a focus on the way that gender, that is the socially constructed differences between men, women and people who identify as gender diverse, results in gendered power relations, which act as social determinants of health.

One important factor underlying gendered differences in health, which is often ignored, is the relationship between gender and power. According to Koester [15], depending of the usage of gender concept, scholars and practitioners have a different perspective on power. For instance, the most common difference is the idea of ‘power over – getting someone to do what you want them to do’ and ‘power to – ability to accomplish an end’ [15]. Feminist literature tends to use the concept of ‘power over’ which frames power in terms of women being oppressed by men [15, 16]. Other scholars suggest that only focusing on gender is too limited because it ignores intersectionality [15–17], an approach that analyzes power expressed in ‘many dimensions, encompassing sexism, racism, class oppression, heterosexism, and other axes of oppression in complex interconnections’ [16].

Is important to understand the relationship between gender and power for health rights. Gender-based inequality in patriarchal societies means differences in access to health services and treatment [18]. Because of unequal gendered power relations, men have more access to structural and institutional power which reside in the forms of access to educational, health, political participation and economic resources and opportunities [17]. These place men in a position of power over women and gender diverse people, which is often cemented by coercive interactions, including violence. These then increase women’s risk for a number of health outcomes. For instance, in South Africa, gendered power inequity in intimate relationships places women at risk of violence, abuse and an increased risk of HIV infection [19]. Gender-based violence illustrates the impact of gender inequality on adverse health outcomes. Numerous researchers [20–23] point to the importance of agency and empowerment on the part of vulnerable communities to redress social inequalities and health inequities.

Gender-based violence is in itself a gendered risk factor for ill health: women are at much higher risk of experiencing gender-based violence than men. Statistics shows that the femicide rate in South Africa is five times higher than the world average [24]. Research conducted by the South African Medical Research Council (MRC) in partnership with Statistics South Africa suggests that one in five partnered women have been assaulted by their partners [25]. At the same time, however, the health care system is often poorly equipped to provide services to survivors of gender-based violence, as is evident in the absence of national guidelines [26]. As a result, women in South Africa who are survivors of gender-based violence often do not receive the necessary or appropriate treatment, including both medical and psychological support [27].

Denton and colleagues [28] argue that gender-attributed differences in health affect men and women differently due to social structure, behavioral and psychosocial determinants. In South Africa, gender inequality impacts on women’s agency to make health-related decisions, for example, in the negotiation of condom use [29, 30]. Social stigma associated with using condoms and women’s socioeconomic disadvantages limits women’s agency in decisions on their sexual and reproductive health [31]. For instance, a woman who suggests condom usage to her partner may be seen as promiscuous [32].

The negative impact of gender inequality on women’s healthcare seems particularly noteworthy given that the majority of healthcare providers and employees in the health sector are themselves women [33]. Women do not only have limited medical and psychological support, but also when seeking healthcare, are often faced with providers who have the power to make choices on their behalf. Health workers’ control over women’s health decision making arises from women’s lack of agency and independence to make their own choices [34]. In addition, health care providers might, in thinking they are promoting healthy choices for women, consciously or unconsciously impose their own preferences and views [34, 35]. These preferences and views may be subconsciously ingrained based on the health workers’ beliefs, own values and work environment or government policies pressure [34, 36]. For instance, provision of family planning services for young women may be resisted by health workers who believe that young women should not be sexually active [29, 30, 36]. This underlines that unequal gendered power relations are a systemic issue and cannot be addressed by individual action alone. Even though a number of civil society organizations supporting the agency of women have developed in South Africa, there are still concerns as to how to address women’s health needs in practice [29]. Unequal gendered power relations not only acts as a social determinant of health, but also influence women’s decision-making agency in the health services [37].

At international level, the UN Economic and Social Council [38, 39] addresses the right to health, and includes specific considerations around gender. In doing so, it mostly focuses on women’s and maternal health, thus highlighting one key aspect of gendered health inequalities. Beyond such biological understandings of the impact of gender on health, however, rights-based approaches also provide the opportunity to locate the relationship between gender and health in wider societal structures and thus address gendered power relations. In framing the relationship between gender and health based on the imperative that all people should be able to exercise and enjoy their rights and participate equally in social, economic and political processes, rights-based approaches widen the understanding of ‘gender’ from health issues that affect specific groups (women) to a critical interrogation of the gendered power relations that act as social determinants of health [40].

In South Africa, non-governmental organisations (NGOs) have a long history of advocating for the right to health, and often also provide specific health services either as stand-alone services or in partnership with government health facilities. For example, the international NGO ‘Medecins sans Frontieres’ provided antiretroviral treatment against HIV before such treatment was available in government health facilities, and ultimately handed over their treatment programme to the Western Cape Department of Health. During the same time period, the NGO ‘Treatment Action Campaign’ relentlessly advocated for access to antiretroviral medication through a combination of widespread treatment literacy programmes, grassroots mobilisation and strategic litigation [41]. Since 1994 however, funding for South African NGOs has continuously decreased [42]. At the same time, NGOs still carry a significant weight in providing health services, especially reproductive health and HIV services [29]. For example, the ‘National Adolescent-Friendly Clinic Initiative’ was launched by the NGO ‘Lovelife’ [29], and ‘Rape Crisis’ in Cape Town is one of the country’s longest-running service providers for support and counselling after sexual and gender-based violence. While the recent years have seen the re-emergence of wider civil society organisations that specifically advocate for reproductive justice (which implicitly challenge gendered power relations; for example, the South African ‘Sexual and Reproductive Justice Coalition’), smaller, service-provision focused NGOs have struggled to maintain their programmes due to funding restraints.

Although research has pointed to the transformative role of NGOs in improving policy and programs regarding women’s health, and directs analyses towards issues of power and power dynamics in health systems [43], many activist movements in health are not explicit in addressing gender or gendered power relations [44–46] or when they do address gender in their work, this might be imposed by donors, international NGOs partners and government [47]. That means organizations advocating for the right to health might focus on specific health issues, but not address the wider patriarchal social structures that underpin gender-related health concerns or inequities. For instance, such narrow foci risk not seeing the impact of rigid patriarchal gender norms on men [48–50]. Men’s values, attitudes and behaviors are influenced by dominant ideas of masculinities - men learn to act in a socially prescribed way, such as being strong, competitive, and are encouraged to take risks and respond with violence (ibid). Such behaviors not only impact men’s health and safety, but also have an impact on women’s health and safety, for example when men perpetrate intimate partner violence against female partners [51].

In addition, NGOs advocating for the right to health that focus on a specfic issue may omit the need to challenge underlying gendered power relations. For instance, the Treatment Action Campaign’s (TAC) legal challenge in the Constitutional Court of South Africa over access to anti retroviral therapy (ART) to prevent mother to child transmission of HIV moved away from women’s rights to access ART to arguing the case on the basis of violating children’s rights to health, thereby undermining arguments related to women’s autonomy (right to choose to use or not the medication) and to women’s reproductive health rights [44].

Given a strong civil society history in SA that uses human rights language, both generally and in the health sector; given that gender is often featured in rights language but may be overlooked in practice; and that gender is often confused with women’s rights, this study sought to explore the question: how aware of gendered power relations are organisations that advocate for the right to health in South Africa, and how does this knowledge impact their work? We do this by focusing on the Learning Network for Health and Human Rights (LN), a network of universities and Civil Society Organisations (CSOs) in South Africa explicitly committed to advancing the right to health, but not explicitly gendered in its orientation. First, we describe the LN. Second, we explore how representatives of LN member organizations understand gender and gendered power relations as conceptual and organizational frames for their organization’s work and for their participation in the LN. Lastly, we discuss how different levels of understanding of the role of gendered power relations impacts on the way the LN advocates for the right to health.

## Methods

A theory-generating, qualitative methodology was used for this study. The study was conducted in July 2014. A purposive sample of LN members was interviewed by the first author of the paper, MFM, using a semi-structured, in-depth interview guide. The study was approved by the Human Research Ethics Committee of the Faculty of Health Sciences at the University of Cape Town (HREC reference number: 400/2014).

### Study setting

The study population was drawn from the members of the Leaning Network for Health and Human Rights (LN). Each of the five civil society organizations and two of the four universities more actively involved in the LN have between 1 and 4 members who are or have participated in the LN structures (described in more detail below). The project was firstly tabled at a LN Executive Committee (EXCO) meeting where all organizations were present to establish organizational buy-in at the outset. Thereafter, individual participants identified from the LN records were approached by phone and email to participate in the study. Attendance at least one LN EXCO meeting was the inclusion criterion to select participants. In total, there were 17 persons who met the inclusion criteria. Participants were enrolled after providing informed consent.

### Study sample

Participants were selected purposively to seek out diversity in terms of roles within the LN, socio-economic status, educational qualifications, professional and employment status. We focused both on those who were in organisational positions, whether employed or elected/appointed, often in leadership positions, and those who were beneficiaries of the organisation’s services or participants in its programmes but not formally identified as organisational leaders. In total, 10 in-depth interviews were conducted with LN participants of whom seven were women and three men. The 10 participants were drawn from 4 of the 5 CSOs and from 1 of the 4 universities involved in the LN. The diversity of the participants enabled us to explore an in-depth understanding of respondents’ perceptions of gender and gendered power relations in their organization’s work and in the work of the LN, as well as how these may have affected their personal development. An overview of participants’ characteristics is summarised in Table 1.

**Table 1:** Participant characteristics

Organisation	Gender	Time in LN
Organisation 1	Woman	6 years
Organisation 1	Man	6 years
Organisation 2	Woman	6 years
Organisation 2	Woman	6 years
Organisation 3	Man	2 years
Organisation 3	Woman	6 years
Organisation 3	Woman	5–6 years
Organisation 4	Woman	4–5 years
Organisation 4	Woman	6 years
University 1	Man	3 years

The in-depth interviews lasted between 45 and 60 min and explored the respondents’ professional background, experience within the LN, understanding and perceptions of gender and gendered power relations, and opinions on the role and impact of gendered power relations in the work of the LN and their own organizations. For instance, we asked about their organization and their role in the organization, how they understood gender and what a ‘gender focus’ meant to them (Additional file 1).

### Data analysis

All interviews were transcribed and analysed manually using thematic analysis. The primary researcher (MFM) read all interviews for key themes and discussed those with the other two authors (LL and AM). After reaching consensus on key themes, all interviews were coded accordingly. To ensure rigor, a sub-set of 4 interviews were coded by another member of the research team (AM) and compared with the interviews coded by the main researcher. Differences were discussed in the team and adjusted after reaching consensus.

## Results

### Describing the learning network and its right-to-health advocacy

The Learning Network for Health and Human Rights (LN) was initiated in 2008 to address the need for collaborative civil society initiatives to advance the right to health and to provide a as a collaborative reflective space for civil society organizations to partner with researchers from two South African universities (University of Cape Town and the University of the Western Cape) and two European universities (Maastricht and Warwick Universities) in facilitating community agency to realise health rights. The five LN Civil Society participants are (a) a grassroots network focusing on empowering women in development, called The Women’s Circle (TWC); (b) the Western Cape branch of Epilepsy South Africa (ESA); (c) Ikamva Labantu (IL), a community development organization in urban Cape Town; (d) Women on Farms Project (WFP), a rural non-governmental organization (NGO) mobilizing and advocating for rural farm women; and (e) the Cape Metro Health Forum (CMHF), an umbrella body for Health Committees in the metropolitan areas of Cape Town. Health Committees were established to be the interface between communities and healthcare facilities in an attempt to concretize community participation in healthcare [52]. Each LN member organisation brings its own frame to right to health work, but at least two of these LN member organizations are explicitly women-oriented and one is explicitly feminist in its methods of work [20].

The LN organizations meet regularly to reflect on their own practice and learning so as to take forward action to improve health and human rights. Participatory action research, capacity building around health rights and strengthening civil society agency for health rights through networking are key features of the LN [53]. LN meetings such as the LN Executive Committee (EXCO), Review and Reflect meetings or LN subgroups are used as a starting point to develop new programs, and to make sure that all LN organizations are on the same page in advocating for health and human rights. Although the LN is attempting to build a collective approach to conceptualising health rights using the different frames brought by member organisations [20], its programmes, training or advocacy have not yet had an explicit focus on gender or gendered power relations.

The action research paradigm within the LN has sought to generate new knowledge about how the right to health can best be realized [54] drawing on traditional African philosophy to model social solidarity as a key element of the right to health [55]. Action research is a collaborative form of research that intimately involves the research participants in the design, evaluation and implementation of research. The LN model also includes various forms of capacity building (for example, see [21]) and, most recently, has begun a long term program focused on capacity building for health committees so as to reinforce the right to health through community participation [56].

### Gender awareness and understanding of gendered power relations

In this section, we explore how representatives of LN member organizations understand gender and gendered power relations as a conceptual and organizational frame for their organization’s work and for their participation in the LN.

Our findings suggest that gendered power relations were understood in terms of individual relationships among the LN members and were framed as stereotypes instead of located in an understanding of systemic power within a patriarchal society. Some female members suggested that ‘gender’ was about a confrontational relationship between men and women and used stereotypes and biological differences between men and women to describe their understanding of gendered power relations. For example, one female participant noted that “[men] are part of the problem” (Participants 1), female respondents largely viewed men as individualists who tend to oppress and exploit women by taking away their “power”. One female member described ‘gender’ as men having the power over women by virtue of the fact that they are biologically different –

“I’m a woman and the fact that I have a vagina […] makes me vulnerable to issues out there, whether it be sexual violence or any kind of abuse – I’m a woman and […] men […] think that they always have the power over a woman especially when it comes to sexual violence, when it comes to rape and all of that, men would use their power to overpower women and the women must always be submissive.” (Participant 9).

The male members offered a different understanding of gendered power relations than the female members, and focused on gender as related to the roles and representation of men and women in society. They mentioned unbalanced representation and “gender oppression” as the cause of gendered power relations. For example, women were not well represented in the government; therefore, according to a male member “men have the power to decide for women and create legislations that better suits their needs” (Participant 7). Male members acknowledged that societal structures should empower women; however, their understanding of women’s empowerment was typically related to increasing job opportunities for women in male-dominated sectors. They believed that women should not be underrepresented in male-dominated sectors; however, women must possess the same competency as men if they want to work in male-dominated sectors. For male LN members, affirmative action approaches to increase the number of women working in male-dominated sectors was problematic because they felt that appointments should be based on qualifications rather than gender.

Similar to the varied understanding of the concept of gendered power relations, there was a disagreement among members on whether or not the LN places a focus on gendered power relations. Some interviewees thought that the LN partner organizations did not really focus on gendered power relations. One participant stated, “within any issues, any training, any of those matters, gender hasn’t been a big thing on the Learning Network’s agenda” (Participant 2). Another member pointed out that because most of the LN members were women, “when we talk, we talk women’s issues. We do not talk about gender-specific issues” (Participant 3).

Other members, on the other hand, were not aware of the possibility of gender invisibility in the LN. One partner offered,

“I think they [LN partner organizations] do a lot around gender issues because one of the main focuses, they also focus on […] maternal health, so the majority would be women in maternal health when it comes to birth and all of those. Whether it’s maternal health, whether it’s access to the clinics and specifically around medication for women, chronic medications, about the health committees, I think they do a lot for women’s issues” (Participant 9).

Confusion about the LN’s focus on “gender issues” therefore relates to different understandings of gendered power relations amongst members. Without properly understanding the meaning and cause of gendered power relations, the LN seems to be caught between its identity and interests. It seems that the woman-led organizations in the LN do not frame the communities’ problems as problems of gendered power relations; but rather as the needs of women in the community and, by doing so, may not challenge underlying gendered power relations and stereotypes.

Respondents argued that, because most of the NGO’s in the LN are considered women’s organizations, the LN therefore has a gender focus. This is consistent with the member’s comment above that, because most members are women, the LN’s focus is reduced to women’s issues. Even though it can be argued that women’s issues are part of a gendered approach, it is important to note that some members made a distinction between women’s issues and ‘gender issues’, and did not feel that the LN had a focus on gendered power relations, despite the fact that its member organizations worked on women’s health concerns.

### Does the understanding of gendered power relations impact the way the learning network advocates for the right to health?

In this section, we explore if the discrepancy in understanding gendered power relations among the LN members impacted the way the LN and its members organisations advocate for the right to health. Without understanding gendered power relations and the impact of unequal power relations on health and human rights, the LN could be potentially gender blind, which in turn shapes the way the LN advocates for the right to health.

In some ways, the assumption that work on gendered power relations was a basic and fundamental aspect of the LN might actually lead LN partners to overlook gender disparities because gender was assumed to be pervasive. As a member pointed out:

“[P]eople in general tend to [indistinct] those differences, they think that they’re natural- and they become invisible – you know, are not problematic, because it’s just normal, it’s just normal life, it’s common sense that the men behaved in this way or that men and women relate in a certain way; we don’t have to remark on it because it’s the same everywhere, it’s normal (Participant 10).”

This pervasive acceptance that men and women behave differently without questioning underlying gendered power relations may create gender invisibility or reinforce lack of gender consciousness.

Most members used their everyday experiences and observations of how men and women behaved in their communities to speak about gendered power relations. These observations, coupled with a recognition of the specific vulnerabilities of women and the reasons therefore, served as justification for the organizations’ work focus. As a member pointed out:

“I think they [men] should be include in continuing the process but I think (pause) sometimes they are part of the problem. They [men] are huge drugs, huge alcohol, you have a lot absent fathers. You have a lot of men that don’t take responsibilities and yes it should be a men’s program where they actually rehabilitated to become men, to become fathers, to it should be a collaborative approach… definitely, but at this point in time women need that special focus and definitely women with disabilities because they are on their own and the can’t wait to rely on the men to be around because of their absence or they walk out” (Participant 1).

The limited understanding of gendered power relations and the fact that most of the LN participants and LN organisations’ members are women, seems to lead the LN’s agenda to women’s issues. One member pointed out that because most of the organizations that are part of the LN are women-led, they end up focusing to improve women’s health and human rights, but do not frame it as feminist work, which would explicitly address the gendered power relationships underlying women’s health disparities:

“I am not a feminist… not what they called a feminist… it’s all go. Whatever man its cool. I then I was WOW. You are right! They are hard core, they don’t back down. It’s like Women, Women, Women. You know. So I’m all women now, but it really raise the right bars… But Yeah, definitely we are dealing with very powerful, very strong community leaders who are women. Who push for women agenda and use women in realizing the needs of the communities” (Participant 1).

Moreover, it seems that even when gendered power relations are explicitly acknowledged in a LN member organisation’s work, it is done within a frame of women’s needs.

“Like I said, on the health programme our main focus would be gender-based violence and doing training with women on gender-based violence. We do weekend trainings which would be two days, but we also go out to the areas where we do specifically on gender-based violence we do capacity building sessions where we talk to the whole of the … community. But within the training specifically we only take women to the training and where we do gender-based violence training (Participant 9).”

Another member, however, felt that this strong focus, whether rooted in a feminist understanding of gendered power relations or not, excluded work with vulnerable men, and that a more nuanced understanding of gendered power relations and vulnerability was lacking in the LN.

“You know, we have messed up gender so much because a feminist will stand up for issues around women and sometimes we actually forget that we [are] not here to speak about issues [only] relevant to us [as women], we are actually here to speak about issues that are relevant to everybody. If there is a human rights violation against men, why can’t I address it? Does it mean that I am not sensitive to men? Does it mean that I do not understand men’s health issues? What is it that I do not understand, what is it that makes me feel that I cannot address the issue around men’s health so for me, I think sometimes we go with the extreme or sometimes we are just very passive with gender issues” (Participant 3).

Before becoming part of the LN, many community members had never been exposed to training or education on human rights or gendered power relations. According to one participant, the LN provided trainings and workshops where they first learned about their human rights and about the concepts of equal rights for men and women. “We got introduced to all sorts of questions which were never answered [*before*], such as rights and issues that could have never been answered” (Participant 6). They were now more confident to continue their work in the communities by spreading their knowledge acquired in the LN trainings and workshops.

The interviews also showed that individual LN members not only felt empowered, but that this also changed organizations’ interests and focus. For example, the women’s organizations that are part of the LN were planning to change their scope. They were not only advocating for women’s rights but are planning to reach out to men. As they have been exposed to the health and human rights concepts, they believed that men should also be included in the outreach. A member suggested that before being exposed to health and human rights concepts, her organization “was just women and the men wanted to attend and I used to say, no, you can’t attend, it’s for women – because we can’t just get things without our organization – so it’s women, women” (Participant 5). However, after being a part of the LN they are now advocating for other individuals; “we went to men: we’re now at the youth, we’re now at [the] Early Childhood Development [sector]” (Participant 5). The participants who advocated for such an egalitarian rights-based approach, however, mostly did not acknowledge the existing gendered power relations in health and healthcare contexts.

## Discussion

In this study, we explored how the LN members understood gendered power relations and how their understanding affected the LN’s work. The results show that there is a discrepancy in knowledge and understanding of gendered power relations between LN members. Having a predominance of female members, or including acknowledgement of disparities among men and women in a mission statement, does not necessarily mean that programmes which aim to challenge unequal gendered power relations are concretized in the organizations’ work. This discrepancy in understanding gendered power relations suggests that they are ‘rendered invisible’ within the LN, which impacts the way the LN advocates for the right to health.

The advantage of using in-depth-interviews was that it made it possible to explore attitudes and understanding deeply. This enabled us to identify discrepancies in the understanding of gendered power relations among LN members, which were (a) gendered power relations as an issue of representation on decision-making structures versus greater equality in decision-making that implies challenging power; (b) gendered power relations as an issue of vulnerability based on sexual/biological differences versus recognising differences arising from how gender is constructed (c) reducing gendered power relations to issues of women versus recognising gendered power relations as being about power.

Those discrepancies, if addressed, could potentially increase the knowledge and awareness of gendered power relations within the LN and improve the LN’s advocacy for health and human rights. Wendoh and Wallace [47] also found that gender concepts, such as the concept of gendered power relations, are usually misunderstood in development agencies and communities. This is often driven by funders’ requirements to adopt gender equality objectives as a condition for funding, which leads NGOs to include gender concepts in their proposal without full understanding, and with little capacity or intention to mainstream gender [47]. However, our findings show that this inconsistency in understanding gendered power relations among the Learning Network partners does not reflect the forced inclusion of gender terminology and objectives as a requirement for funding but instead may reflect insufficient knowledge and training around gender and power, or the lack of an explicitly feminist lens to the work of health and human rights.

Understanding gendered power relations is fundamental for the LN, as a human rights-based network, to continue to inclusively advocate for health and human rights [57]. Despite progressive legislation, South Africa has profound gender inequality; women in South Africa are more likely to suffer health and rights violations, and there is emerging evidence that transgender and gender diverse people are systematically excluded from health policy and healthcare delivery [58, 59]. Our findings suggest that the member organizations of the LN push for the empowerment of women. However, our findings also suggest that focusing only on women’s rights may not challenge the underlying gendered power relations or address the marginalisation of gender diverse people’s health concerns. Men are also negatively impacted by gendered power relations and should being involved in gender transformation programmes [48–50, 60].

The LN is an organization that is concerned about inequalities in the health system, such as lack of community participation and violations of health rights. Even though most of its members are women, one cannot assume that this translates into an awareness of gendered power relations among the LN’s members and in the LN’s work. Researchers argue that even individuals who work for agencies advocating for gender equality might still suffer from gender blindness [44–47]. There is therefore a need to explore gendered power relations explicitly within the LN’s structures and programmes; otherwise the LN will remain essentially gender-blind. However, understanding gendered power relations is important so that organizations and health advocates can move beyond stereotypes about men and women and can understand that gendered power relations are caused by more than biological differences, rather, by patriarchal society [61]. Increasing LN members’ gender literacy and understanding of feminist approaches to health rights work could help for participatory health decision-making to be more equalized among the LN organizations. Being aware of gendered power relationships can also help partners to recognise and address these structural inequalities in their work.

Many researchers have suggested that civil society plays a fundamental role in advocating for human rights and gender equality [29, 43, 62]. Moreover, organizations such as the LN members can influence legislative and policy changes. Therefore, to continue to advocate for health and human rights, we argue that gender literacy, and specifically knowledge around gendered power relationships, should be included in health and rights trainings as a specific focus, because there is a divergence in understanding of what gendered power inequalities are and how they affect health. Snow [63] argues that it is extremely important to understand the difference between sex and gender and the complex inter-relationship between the two concepts that impact on individuals’ health and on health inequalities. She suggests that only by understanding how gender and sex play a role on health inequalities can intervention be made to improve individuals’ health [63].

In the results, interviewees mentioned how useful other LN trainings on health and human rights had been for them and their communities. Therefore, we suggest that the LN can improve its existing health and rights programmes by creating a tool-kit that explicitly explores and explains (1) sex vs gender; (2) gendered power relationships in patriacrchal societies; (3) the relationship of women’s health/rights to gender and health/rights; and (4) gender equality as more than just representation but equality in decision making and power. This tool-kit could be used in trainings as a starting point and should translate feminist understanding of gendered power relations into practice using language that the community can relate to and understand. The LN should not only make sure members have participated in trainings that explain gender power relations, but it should also be the LN’s responsibility to ensure the realisation of the Right Health is able to address gendered power relationships and include the concerns of transgender and gender diverse individuals.

## Conclusion

The overall results show that there is a discrepancy in the knowledge of gender and gendered power relations between LN members. Our findings are important beyond the LN, as they show that even organizations that state a gender-based focus might be unaware of the possibility of gender invisibility. Other networks/groups, particularly human rights groups, should spend time reflecting on how their members perceive gendered power relations and how a discrepancy could potentially affect the organization’s mission, goals and programmes. We suggest that, in their trainings, civil society organisations must illustrate how biological notions of sex and social construction of gender lead to gendered power relations and play a role in health inequalities. To keep the health and human rights’ agenda successfully moving forward and for gender mainstreaming initiatives to be effective, civil society in South Africa and elsewhere must acknowledge the importance of gendered power relationships in advocating for gender equality. The ‘Diabetes Prevention and Control’ in Mexico, in which gender-specific brochures were used to address stereotypes that result in risky behavior for men and women differently, and ‘Primary health care with a gender approach’ (Star Health Services) in Bolivia, which provided care for migrant and Aymara indigenous women are successful examples how gender has been mainstreamed to ensure a more inclusive health [64]. It is by increasing the understanding of gendered power relations that we will be able to better advocate for a reduction of health disparities based on gender.

## Additional file

Additional file 1: Interview Guide. (DOCX 14 kb)

## Abbreviations

CMHF: Cape Metro Health Forum
CSOs: Civil Society Organisations
ESA: Epilepsy South Africa
EXCO: LN Executive Committee
IL: Ikamva Labantu
LN: The Learning Network for Health and Human Rights
TAC: Treatment Action Campaign
TWC: The Women’s Circle
WFP: Women on Farms Project

## Glossary

Gender: The socially constructed characteristics of individuals; usually associated with “being a man and woman”. For a long time, gender was assumed to be binary (ie. women and men), however a more contemporary understanding of gender is that it exists on a continuum, with women and men being on opposite ends, as well as gender diverse, including transgender people.
Sex: Biological characteristics that define whether a person is female or male. These include external and internal genitalia, as well as hormones.
Gender norms: Ideas how men and women should ‘typically’ behave. These influence what is considered ‘acceptable’ behaviour for women or men in society. Gender norms are specific to both geographical location and historical moment of a society. Often these are enforced through social policing, ranging from social pressure to social exclusion to violence.
Gender roles: Social and behavioural norms attributed to individuals according to their gender. Closely linked to gender norms, and often stereotypical.
Gendered power relations: Unequal social relations of power between gendered persons, most often men and women, due to patriarchy and the resulting unequal distribution of wealth, resources and social power. Based on Connell’s relational theory of gender and power [17].
Gender- based violence: Physical, sexual or psychological violence against a person or group of people based on their gender. Most often perpetrated along the power differential, i.e. by men against women or transgender people.
