# Supplementary material for: Missing knowledge of gendered power relations among non-governmental organisations doing right to health work: a case study from South Africa
Source: BMC Int Health Hum Rights. 2018 Aug 30;18:33. doi: 10.1186/s12914-018-0172-4 (PMC6117970; doi:10.1186/s12914-018-0172-4)
Supplement: Supplementary file 1 — Interview Guide. (DOCX 14 kb) [file 12914_2018_172_MOESM1_ESM.docx]

**Additional File 1 - Interview Guide**

*Instructions to interviewer: The interview will start with the researcher introducing herself and an explanation of the research project. The voluntary nature of the process will be emphasised and the participant will be requested to sign a consent form. Issues of confidentiality will be discussed. Work through the Information Sheet with the respondent and the complete the consent form if the respondent is happy to participate:*

Broad Questions:

1. As a way of introduction, can you tell me a little about your organisation and your role in the organisation?
   1. How long have you been in the organisation?
   2. *Probe what is the respondent’s position, whether and how has it changed over the past 5 or 6 years.*
   3. What is the mission of the organisation?
2. Does your organisation make gender an explicit focus of its work? If so, how so? Can you give examples?
   1. *Probe how they understand gender and a gender focus e.g.* “So how do you under a gendered approach to …xxx”
   2. *Tease out what they suggest as examples to see if they really are gender issues*
   3. Since your involvement with the Learning Network, has there been any change in the way your organisation deals with gender?
      *Probe both inward (internal to the organisation) and external (with the clients or constituency they serve)*
3. When did you first get involved in the LN?
   1. What was your motivation for getting involved?
   2. Has your involvement in the LN changed the way you work?
   3. Has your involvement in the LN changed the way you think about gender issues?
4. Do you think the LN is a space where gender issues are adequately addressed?
   1. Do you think the LN does enough or too little or too much about gender issues?
   2. Do you think it is safe space to discuss gender issues?
5. Do you feel the LN has helped your own development? Do you think the fact that you are a <woman/man> had influenced how you have developed through the LN?

*At the end of the discussion, the researcher will thank the respondent for their contribution. She will also indicate that the research will be collated and written up in a report. A copy of the transcript will be made available to the respondent if they wish to check its accuracy and the report will be discussed in the LN meetings, and will be available for the individual on their request.*
